# Mechanistic and Therapeutic Insights into Nrf2-Mediated Redox Regulation in Periodontitis

**DOI:** 10.3390/antiox15010072

**Published:** 2026-01-06

**Authors:** Satoshi Wada, Hiroyuki Nakano, Yasuhisa Sawai, Yota Yamauchi, Miho Hasumoto, Eiji Mitate, Noboru Demura

**Affiliations:** Department of Oral and Maxillofacial Surgery, Kanazawa Medical University, Kahoku 920-0293, Ishikawa, Japan

**Keywords:** periodontitis, oxidative stress, redox signaling, Nrf2/Keap1 pathway, pyroptosis, ferroptosis, bone remodeling, host-modulation therapy

## Abstract

Periodontitis is a chronic non-communicable inflammatory disease in which oxidative stress plays an important role in tissue destruction and alveolar bone loss. Excessive production of reactive oxygen species disrupts redox homeostasis, activates inflammatory signaling pathways, and promotes regulated cell death processes such as pyroptosis and ferroptosis. The Nrf2/Keap1 pathway is a key regulator of antioxidant defense and cellular adaptation to redox imbalance. Impaired Nrf2 signaling has been associated with enhanced oxidative injury, NF-κB and NLRP3 inflammasome activation, osteoclast-driven bone resorption, and reduced regenerative capacity in periodontal tissues. Experimental studies suggest that Nrf2 activation can restore the redox balance and attenuate inflammation and bone destructive responses in a periodontal model. Moreover, therapeutic approaches involving phytochemicals, microbial-derived metabolites, and redox-responsive biomaterials have been reported to influence Nrf2-related signaling in experimental settings. However, the majority of the available evidence is derived from in vitro or animal studies, and the relevance of these findings to clinical periodontitis remains to be established. This review summarizes the current advances linking oxidative stress, redox signaling, cell death pathways, and bone remodeling with Nrf2 dysfunction in periodontitis and outlines the key mechanistic insights while highlighting the existing knowledge gaps.

## 1. Introduction

Periodontal disease is a common chronic inflammatory disorder of the oral cavity that progresses from plaque-induced gingivitis to irreversible destruction of the periodontal supporting tissues [1,2]. If left untreated, inflammation extends to the periodontal ligament, cementum, and alveolar bone, leading to attachment loss and, in severe cases, tooth exfoliation [1,2,3]. Disease progression is characterized by alternating phases of active breakdown and partial remission, reflecting a multifactorial and self-perpetuating process driven by microbial dysbiosis, host immune responses, and systemic modifiers [2,4,5].

Dysbiotic biofilms dominated by Gram-negative anaerobes such as *Porphyromonas gingivalis* and *Aggregatibacter actinomycetemcomitans* activate innate immune signaling and promote the release of pro-inflammatory mediators, including interleukin-1β (IL-1β), tumor necrosis factor-α (TNF-α), and matrix metalloproteinases (MMPs) [4,5]. These pathogens also stimulate the excessive production of reactive oxygen species (ROS) by neutrophils and macrophages, contributing to oxidative stress and amplifying downstream inflammatory signaling. Chronic overproduction of these mediators induces connective tissue destruction and alveolar bone resorption, reinforcing the cycle of inflammation and tissue breakdown [6,7,8]. Clinically, periodontitis presents with bleeding on probing, periodontal pocket formation, attachment loss, and radiographic evidence of bone loss [9].

Beyond its local manifestations, periodontitis contributes to systemic oxidative and inflammatory stress and is epidemiologically linked to several non-communicable diseases, including cardiovascular disease and diabetes mellitus; elevations in circulating C-reactive protein have been consistently reported [10]. Excessive generation of ROS plays an important role in amplifying inflammatory signaling and accelerating periodontal tissue breakdown, highlighting oxidative stress as a key pathogenic driver [6,7]. The maintenance of redox homeostasis, therefore, represents a key determinant of disease severity and progression.

Among the molecular regulators of antioxidant defense, the nuclear factor erythroid 2-related factor 2 (Nrf2) pathway functions as a master transcriptional controller of cytoprotective and detoxification responses [11,12]. Once released from Keap1, Nrf2 translocates to the nucleus and induces antioxidant enzymes such as heme oxygenase-1 (HO-1), NAD(P)H: quinone oxidoreductase-1 (NQO1), and glutathione-related proteins. These responses protect the periodontal tissues from oxidative injury, yet alterations in Nrf2 activity have been reported in oral neutrophils, periodontal ligament stem cells (PDLSCs), and diabetes-associated periodontitis, suggesting impaired redox adaptation in disease states [13,14,15].

Recent evidence also indicates that Nrf2 activity is regulated by its transcriptional repressor BTB and CNC homology 1 (Bach1), which competes for binding to antioxidant response elements (AREs) and suppresses Nrf2-dependent gene expression under basal conditions [16]. While oxidative stress promotes Bach1 inactivation and facilitates Nrf2-mediated antioxidant response, dysregulated Bach1 activity may further lower the antioxidant capacity and contribute to tissue damage.

In this review, we summarize the current evidence on the role of Nrf2 signaling in periodontal disease—with emphasis on its interaction with oxidative stress, Bach1-related regulatory mechanism, regulated cell death pathways (ferroptosis/pyroptosis), and bone remodeling—and discuss the therapeutic relevance of targeting Nrf2 in host-modulation strategies.

## 2. Nrf2/Keap1 Pathway: A Central Regulator of Cellular Redox Homeostasis

Nrf2 is a master transcription factor that orchestrates cellular defense against oxidative and electrophilic stress. Under basal conditions, Nrf2 is sequestered in the cytoplasm by Kelch-like ECH-associated protein 1 (Keap1), a substrate adaptor of the Cullin-3 E3 ubiquitin ligase complex that promotes Nrf2 ubiquitination and proteasomal degradation [11]. Keap1 functions as a redox sensor: specific cysteine residues (notably Cys151, Cys273, and Cys288) undergo covalent modification in response to oxidants or electrophiles, suppressing Nrf2 ubiquitination and allowing newly synthesized Nrf2 to accumulate, translocate to the nucleus, and heterodimerize with small Maf proteins [11]. The Nrf2–Maf complex then binds AREs and induces cytoprotective genes—including HO-1, NQO1, superoxide dismutase (SOD), catalase (CAT), and glutathione-synthesizing enzymes—thereby restoring the intracellular redox balance [11]. Beyond periodontal tissues, dysregulation of the Nrf2/Keap1 pathway has been implicated in a wide range of diseases, including cancers, where aberrant Nrf2 activation contributes to tumor progression, metabolic reprogramming, and therapy resistance [17,18].

Beyond its canonical antioxidant function, Nrf2 intersects with metabolic and inflammatory signaling. Crosstalk with PI3K/Akt and MAPK pathways contributes to stress adaptation, while Nrf2 counteracts inflammatory amplification by antagonizing nuclear factor kappa-light-chain-enhancer of activated B cells (NF-κB)-dependent transcription and restraining NLR family pyrin domain-containing protein 3 (NLRP3) inflammasome activation, thus linking redox control to immune homeostasis [12,19,20]. Acting as an important regulator by integrating cellular stress responses, Nrf2 coordinates adaptive programs that preserve tissue integrity across multiple organs and chronic disease contexts, including cardiovascular, metabolic, and inflammatory disorders [11,12]. Dysregulation of this pathway, therefore, provides a unifying framework linking redox imbalance to chronic inflammatory tissue injury relevant to periodontitis pathogenesis. Although primarily regulated by Keap1, Nrf2 activity is also influenced by Bach1; this aspect is further addressed in Section 4.5 [16,21].

## 3. Oxidative Stress and the Dysregulation of Nrf2 Signaling in Periodontitis

Oxidative stress plays a pivotal role in the pathogenesis of periodontitis, linking microbial challenge to host-mediated connective tissue and alveolar bone destruction. Periodontitis is characterized by the disruption of redox homeostasis arising from the excessive production of ROS and insufficient antioxidant defenses [6]. During disease progression, polymorphonuclear neutrophils—predominant immune cells recruited to the gingival crevice—generate large quantities of ROS in response to bacterial challenge [7]. However, sustained or dysregulated ROS release perturbs the redox balance and induces the oxidative modification of lipids, proteins, and nucleic acids, leading to cellular dysfunction, apoptosis, and the degradation of periodontal ligaments and the bone matrix [7]. Moreover, ROS act as upstream activators of NF-κB and the NLRP3 inflammasome, thereby amplifying inflammatory signaling and accelerating tissue destruction, resulting in a self-perpetuating cycle of inflammation and oxidative injury [8,22].

The clinical and experimental evidence support this mechanism. Patients with periodontitis display reduced systemic and local antioxidant capacity, including decreased total antioxidant capacity (TAOC) and diminished SOD and glutathione peroxidase (GPx) activities [23,24,25,26], accompanied by elevated malondialdehyde (MDA), a marker of lipid peroxidation [24,26,27]. Notably, impaired Nrf2 signaling has been increasingly recognized as a feature of this oxidative imbalance. Gingival tissues and oral neutrophils from patients with severe periodontitis exhibit reduced Nrf2 expression and activity [28], while Nrf2-deficient mice show enhanced oxidative stress, increased inflammatory mediator expression, and aggravated alveolar bone loss in experimental periodontitis models [13]. Collectively, these findings support the view that reduced Nrf2 activity contributes to redox imbalance and inflammatory tissue destruction in periodontitis, providing a rationale for Nrf2-targeted host modulation strategies. However, the strength of the available evidence varies across clinical and experimental studies.

## 4. Protective and Regulatory Roles of Nrf2 in Periodontitis

### 4.1. Nrf2 and Apoptosis Regulation in Periodontitis

Apoptosis is activated in gingival fibroblasts, epithelial cells, neutrophils, and macrophages in response to periodontal microbial stimuli and pro-inflammatory cytokines [29]. Increases in TUNEL-positive cells in inflamed periodontal tissues have been reported to correlate with collagen degradation and alveolar bone loss [30]. While apoptosis contributes to the removal of damaged cells and may support inflammatory resolution, persistent or excessive apoptosis can disrupt the reparative homeostasis and compromise periodontal tissue integrity [31]. Consistently, the dysregulated expression of apoptosis-related genes, including CASP2, CASP8, CASP3, BCL2, and TP53, has been detected in periodontitis lesions [32,33].

Oxidative stress is a major upstream trigger of apoptotic signaling in periodontal cells. Impaired Nrf2 activity may further exacerbate oxidative damage, increasing the susceptibility to apoptosis and hindering periodontal repair [15]. Conversely, Nrf2 activation suppresses pro-apoptotic mediators (caspase-3, caspase-9, Bax) and enhances anti-apoptotic Bcl-2, thereby improving cell survival under excessive oxidative stress [14]. However, the available evidence is largely based on experimental models, and it is still unclear whether Nrf2 impairment is causative or secondary to chronic inflammation and dysbiosis. In addition, because antioxidant gene transcription is also shaped by the Nrf2-Bach1 balance at AREs, future studies should clarify how this regulatory axis influences apoptosis and tissue outcomes in periodontitis. Taken together, these findings suggest the protective role of Nrf2-mediated redox regulation in limiting excessive oxidative stress-driven apoptosis, although important mechanistic and translational questions remain.

### 4.2. Pyroptosis in Periodontitis

Pyroptosis, an inflammatory form of programmed cell death, is primarily mediated through NLRP3 inflammasome activation, caspase-1–dependent cleavage of gasdermin D (GSDMD), and the release of IL-1β and IL-18 [34,35]. Notably, pyroptosis can also have a protective function in acute infection; however, in the context of chronic periodontal inflammation, sustained inflammasome activation is thought to exacerbate tissue destruction. This pathway amplifies periodontal inflammation, promotes osteoclastogenesis, and accelerates alveolar bone destruction [36]. ROS serve as key upstream triggers that potentiate inflammasome assembly, contributing to a feed-forward oxidative inflammatory cycle [8]. Nrf2 activation counteracts this process by inducing antioxidant enzymes such as HO-1 and NQO1, thereby inhibiting ROS-dependent NLRP3–caspase-1 activation [37,38]. Nrf2 deficiency leads to heightened pyroptosis and aggravated bone loss in experimental periodontitis [39]. Conversely, Nrf2 activators—including sulforaphane, resveratrol, and other natural polyphenols—have been reported to suppress inflammasome-driven pyroptosis [40,41,42], and the vitamin D analog ED-71 similarly reduces GSDMD-mediated pyroptotic injury via Nrf2/HO-1 signaling [43]. Thus, Nrf2-mediated regulation of the ROS–NLRP3 axis appears to play an important role in limiting pyroptotic periodontal damage.

### 4.3. Ferroptosis in Periodontitis

Ferroptosis is an iron-dependent and lipid peroxidation-driven form of regulated cell death and is increasingly recognized as causing periodontal tissue destruction [44]. While ferroptosis has also been implicated in host defense and stress adaptation; however, under chronic inflammatory conditions such as periodontitis, dysregulated ferroptotic signaling is thought to contribute to tissue injury. The ferroptosis inducer erastin aggravates bone loss, whereas interleukin-17 (IL-17) attenuates this effect by enhancing osteogenesis through STAT3–Nrf2-mediated upregulation of GPX4 and SLC7A11 [45]. Nrf2 restricts ferroptosis by promoting antioxidant enzymes, such as HO-1, NQO1, and GPX4, and by suppressing excessive ROS accumulation [44,46]. In periodontal ligament stem cells, the antimicrobial peptide bomidin inhibits TNF-α-induced ferroptosis via Keap1 degradation and Nrf2 stabilization [47]. In vivo, Ferrostatin-1 reduces inflammation and bone loss, while apical periodontitis tissues exhibit downregulation of Nrf2, GPX4, and FSP1 accompanied by ferroptotic macrophage activation [48]. Collectively, these observations indicate that modulation of the Nrf2–GPX4–FSP1 axis may represent a potential approach to limiting ferroptosis-associated periodontal tissue injury.

### 4.4. Crosstalk Between Pyroptosis and Ferroptosis

Accumulating evidence indicates that pyroptosis and ferroptosis are not independent cell death programs but are interconnected through shared redox-sensitive and inflammatory signaling pathways. ROS production represents a key point of convergence, as ROS not only promotes lipid peroxidation and ferroptotic cell death but also facilitates inflammasome assembly and pyroptotic activation. Therefore, in periodontal tissues, sustained oxidative stress has the potential to simultaneously amplify ferroptosis and pyroptosis, creating a self-reinforcing cycle of inflammation and tissue damage [8,49].

Recent studies suggest that ferroptotic cells can exacerbate inflammatory responses by releasing damage-associated molecular patterns (DAMPs) and lipid peroxidation products, which in turn potentiate inflammasome activation and downstream pyroptotic signaling. Conversely, inflammasome-driven cytokines such as IL-1β and IL-18 may further disrupt redox homeostasis and sensitize the surrounding cells to ferroptosis, particularly under chronic inflammatory conditions. These observations support a bidirectional interaction between ferroptosis and pyroptosis that contributes to the persistence of periodontal inflammation and progressive alveolar bone loss [20,37].

Nrf2 signaling occupies a central regulatory position in this crosstalk by modulating both oxidative stress and inflammatory signaling. Through the induction of antioxidant and redox-buffering programs, Nrf2 limits ROS accumulation, thereby restraining ferroptotic lipid peroxidation and dampening inflammasome activation [19,20]. However, Nrf2-dependent transcriptional responses are not governed by Nrf2 alone but are influenced by its interplay with transcriptional repressors such as Bach1 in antioxidant response elements. Disruption of this regulatory balance may therefore shift the periodontal microenvironment toward a pro-oxidant and pro-inflammatory state, creating conditions that are permissive for the activation of ferroptosis and pyroptosis [16,21].

Importantly, the functional consequences of this crosstalk appear to be context-dependent. While transient activation of inflammatory cell death pathways may contribute to host defense and microbial control during acute infection, persistent dysregulation of redox and inflammasome signaling in chronic periodontitis is more likely to drive tissue destruction rather than resolution. Collectively, the available evidence suggests the importance of coordinated redox–inflammatory regulation in determining cell death outcomes and suggests that therapeutic strategies aimed at restoring the Nrf2-Bach1 balance and redox homeostasis may simultaneously attenuate both ferroptosis and pyroptosis in periodontal disease.

### 4.5. Bach1 as a Context-Dependent Regulator of Nrf2 Signaling in Periodontitis

Bach1 is a transcriptional repressor that competes with Nrf2 for binding to AREs, thereby limiting the expression of antioxidant and cytoprotective genes under basal or pro-oxidant conditions [11,16]. Through this antagonistic interaction, Bach1 functions as a critical modulator of cellular redox homeostasis, preventing excessive or prolonged activation of Nrf2-driven antioxidant responses.

In periodontal tissues, dysregulation of the Nrf2–Bach1 balance may have important pathological implications. Experimental studies have shown that enhanced Bach1 activity suppresses antioxidant enzyme expression, leading to increased intracellular ROS accumulation and amplification of inflammatory and osteoclastogenic signaling. Conversely, inhibition of Bach1 has been reported to reinforce antioxidant capacity, attenuate ROS-dependent signaling cascades, and suppress osteoclast differentiation, resulting in reduced inflammatory bone destruction in vivo [16,21]. Consistent with this concept, recent experimental evidence demonstrates that knockdown of Bach1 under inflammatory conditions restores the osteogenic capacity of periodontal ligament cells and promotes periodontal bone regeneration [50].

Notably, the regulatory role of Bach1 appears to be highly context-dependent. While transient Bach1-mediated restraint of Nrf2 activity may be necessary to fine-tune redox signaling and immune responses, persistent or excessive Bach1 activation in chronic inflammatory conditions, such as periodontitis, is more likely to exacerbate oxidative stress, inflammation, and tissue destruction. In addition to these effects, Bach1 has also been implicated in the regulation of ferroptosis by modulating cellular iron handling and the redox balance.

Consistent with these observations, studies in other bone-related disorders have shown that Bach1 suppresses osteoblast survival and differentiation while promoting osteoclastogenesis under oxidative or inflammatory stress, highlighting a conserved role of Bach1 in the redox-sensitive regulation of bone remodeling [51,52].

Together with evidence from the apoptosis, pyroptosis, and ferroptosis pathways, these observations position Bach1 as a key determinant of redox-dependent cell fate decisions in periodontitis. Disruption of the Nrf2–Bach1 equilibrium may shift the periodontal microenvironment toward a pro-oxidant and pro-inflammatory state that favors pathological cell death and bone resorption. Recent experimental studies suggest that the pharmacological targeting of Bach1, including approaches that promote its degradation, may attenuate oxidative stress and periodontal inflammation in vivo [53,54].

### 4.6. Nrf2 and Inflammatory Factors in Periodontitis

In the inflammatory microenvironment of periodontitis, Nrf2 functions as an important regulator of oxidative and inflammatory responses. Upon activation, Nrf2 translocates to the nucleus and induces antioxidant enzymes such as HO-1 and NQO1, while also suppressing the transcription of pro-inflammatory mediators, including *IL1B* and *IL6* [55]. Furthermore, Nrf2 inhibits NF-κB signaling and restrains NLRP3 inflammasome activation, thereby limiting inflammation-associated cell death and tissue injury [19,20].

Experimental studies demonstrate that the pharmacological activation of Nrf2 reduces cytokine production, attenuates oxidative damage, and limits periodontal tissue destruction, whereas these protective effects are markedly diminished in Nrf2-deficient models [39,56,57,58,59]. Collectively, these findings indicate that Nrf2 can attenuate the feed-forward interaction between oxidative stress and inflammation, thereby contributing to the maintenance of periodontal immune homeostasis.

### 4.7. Role of Nrf2 in Bone Metabolism in Periodontitis

In a periodontitis, there is excessive osteoclast-mediated bone resorption under inflammatory and oxidative conditions [7,60,61]. The receptor activator of NF-κB ligand (RANKL) is the central mediator of osteoclastogenesis, and oxidative stress plays a pivotal role in amplifying RANKL-induced differentiation signals [62,63,64]. Nrf2 activation contributes to the restoration of the redox balance by inducing HO-1, NQO1, and other antioxidant enzymes, thereby attenuating osteoclast differentiation and bone resorption [64,65,66]. In addition, Nrf2 can antagonize NF-κB signaling and reduce RANKL expression within inflamed periodontal tissues, further limiting osteoclast activity [64,67,68,69,70].

Beyond osteoclast regulation, Nrf2 supports osteoblast differentiation by mitigating oxidative inhibition and limiting apoptosis, thereby promoting bone formation and matrix homeostasis [71,72,73,74,75]. Consistently, Nrf2-deficient mice have displayed increased osteoclast numbers, impaired osteoblast activity, and severe alveolar bone loss in experimental periodontitis [13,15,39]. The pharmacological activation of Nrf2 has been shown to suppress osteoclastogenesis and protect against periodontal bone destruction in vivo [57,76,77,78,79]. Collectively, these findings indicate that Nrf2 contributes to skeletal homeostasis by coordinating redox regulation between osteoclast and osteoblast activity. However, excessive and prolonged Nrf2 activation may disrupt physiological bone turnover, highlighting the importance of context-dependent regulation [80,81]. Importantly, these findings indicate that Nrf2 activation is not uniformly beneficial and that both insufficient and sustained activation can negatively affect bone homeostasis.

## 5. Therapeutic Implications: Targeting Nrf2 in Periodontal Disease

Therapeutic strategies for periodontitis remain constrained by the limitations of conventional antimicrobial approaches, and the effective modulation of host oxidative stress continues to represent a major clinical challenge. In this context, natural compounds that activate Nrf2, a master regulator of antioxidant defense, have emerged as promising adjunctive strategies for restoring the redox balance and attenuating inflammation. Accumulating evidence indicates that a wide range of phytochemicals, bioactive metabolites, and engineered nanomaterials can activate Nrf2 signaling while concurrently suppressing NF-κB-mediated inflammatory pathways, thereby alleviating oxidative injury, reducing pro-inflammatory cytokine production, and contributing to alveolar bone preservation.

**Figure 1:**
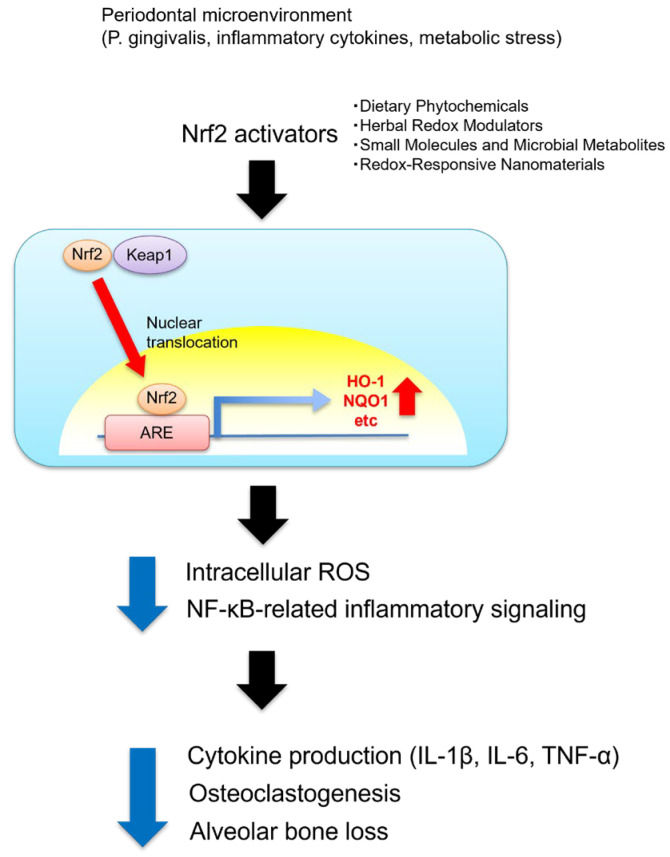
A schematic illustration of experimentally reported Nrf2-mediated redox regulation in periodontitis. Nrf2 activators, including dietary phytochemicals, herbal redox modulators, small molecules and microbial metabolites, and redox-responsive nanomaterials, promote the dissociation of Nrf2 from Keap1 and facilitate its nuclear translocation. Nuclear Nrf2 binds to ARE, inducing the expression of cytoprotective enzymes such as HO-1 and NQO1. Activation of the Nrf2/ARE pathway reduces the intracellular ROS levels and suppresses NF-κB signaling, thereby decreasing pro-inflammatory cytokine production (e.g., IL-1β, IL-6, TNF-α), inhibiting osteoclastogenesis, and ultimately attenuating alveolar bone loss in periodontitis. The pathways illustrated are primarily supported by experimental evidence from in vitro and animal studies, and their relevance to human periodontitis has not yet been fully established.

Figure 1 summarizes the therapeutic landscape of Nrf2 activation in periodontitis. Various classes of Nrf2 activators—including dietary phytochemicals, herbal redox modulators, small molecules, microbial metabolites, and redox-responsive nanomaterials—activate Nrf2/ARE signaling, suppress NF-κB-driven cytokine release, and promote osteogenic and reparative responses. Collectively, these mechanisms contribute to the maintenance of redox homeostasis and the preservation of alveolar bone under oxidative inflammatory conditions (Figure 1). In this context, emerging evidence suggests that relief of Bach1-mediated transcriptional repression may represent an additional regulatory layer shaping Nrf2-dependent antioxidant responses in periodontal tissues. The following sections highlight recent advances in dietary, herbal, and engineered Nrf2 modulators and present their therapeutic potential in mitigating periodontal inflammation and promoting periodontal tissue regeneration.

## 6. Natural Compounds and Engineered Redox Modulators Targeting Nrf2/NF-κB Signaling in Periodontitis

In this section, we classify Nrf2-targeting redox modulators investigated in experimental periodontitis into four major categories: (1) dietary phytochemicals, (2) herbal and traditional medicine-derived metabolites, (3) novel small molecules including lipid-derived and microbial metabolites, and (4) engineered redox-responsive delivery platforms. Rather than providing an exhaustive catalog, this we focus on representative compounds with defined mechanistic evidence linking redox regulation to inflammatory control and alveolar bone preservation. Importantly, limitations related to the experimental design, bioavailability, and translational relevance are explicitly discussed to avoid overgeneralization of the therapeutic potential.

### 6.1. Representative Redox-Active Dietary Phytochemicals Investigated in Experimental Periodontitis

Representative dietary phytochemicals targeting the Nrf2/NF-κB axis are summarized in Table 1. These compounds have been reported to attenuate periodontal inflammation and oxidative injury by inducing Nrf2/ARE-dependent cytoprotective programs while suppressing NF-κB-driven inflammatory signaling and inflammasome activation [6,8,19,20]. However, most of the evidence remains preclinical, and the reported effects vary depending on the experimental design, formulation, and disease models. Accordingly, these agents are appropriately regarded as mechanistic prototypes rather than established periodontal therapeutics [6,7,39].

Sulforaphane. Sulforaphane, an isothiocyanate from cruciferous vegetables, has been reported to restore redox balance and modulate inflammation in periodontal disease. Notably, sulforaphane improves neutrophil redox homeostasis and reduces hyperactive oxidative responses in chronic periodontitis-associated neutrophils, supporting Nrf2-centered host modulation [82]. In gingival epithelial cells, sulforaphane can cooperate with adjunct bioactive fractions to enhance Nrf2-driven antioxidant signaling [83]. Importantly, recent evidence further demonstrates that sulforaphane suppresses *Porphyromonas gingivalis* LPS-induced inflammatory responses in human gingival fibroblasts and attenuates ligature-induced periodontitis in rats via Nrf2-dependent antioxidant defense, leading to reductions in oxidative stress, mitochondrial dysfunction, osteoclast formation, and alveolar bone loss [77]. In addition, sulforaphane may inhibit inflammasome activation through mechanisms that are not exclusively dependent on Nrf2, highlighting the pathway complexity and the need for careful mechanistic attribution [40].

Quercetin. Quercetin, a dietary flavonoid abundant in fruits and vegetables, exerts antioxidant, anti-inflammatory, and antibacterial effects in periodontal tissues [84,85,86,87]. It protects human periodontal ligament cells from oxidative injury by activating the Nrf2/HO-1 pathway and upregulating antioxidant enzymes (HO-1, NQO1) [84]. Quercetin also modulates the p53/p21 axis, preventing oxidative stress-induced apoptosis and senescence [84]. In vivo, it normalizes SOD, CAT, and ceruloplasmin activities, reduces MDA accumulation, and attenuates alveolar bone resorption [85,86]. Ligature-induced models reveal decreased iNOS, MMP-8, and caspase-3 with increased TIMP-1, supporting anti-resorptive and pro-regenerative effects [86]. Moreover, in *Aggregatibacter actinomycetemcomitans*-induced periodontitis, quercetin downregulated IL-1β, TNF-α, IL-17, RANKL, and ICAM-1 without altering the bacterial load, demonstrating immunomodulatory properties [87]. At present, the clinical evidence for quercetin in periodontitis is limited and heterogeneous, and its low oral bioavailability has led to increasing interest in topical and delivery-based strategies [88,89]. Overall, quercetin appears to function primarily as an adjunctive host-modulatory compound, with its clinical relevance likely influenced by the formulation and periodontal-specific trial design.

Isorhamnetin. Isorhamnetin, a flavonoid isolated from *Hippophae rhamnoides* fruit, decreases PGE_2_, NO, IL-6, and IL-8, inhibits NF-κB in gingival fibroblasts, and upregulates Nrf2/HO-1; Nrf2 knockdown reverses these effects [90]. In macrophages stimulated with *Prevotella intermedia* LPS, isorhamnetin markedly suppressed IL-6 production and mRNA expression while inducing HO-1 at both transcriptional and protein levels [91]. The HO-1 inhibitor tin protoporphyrin IX abolished this effect, while isorhamnetin also inhibited NF-κB p50 nuclear translocation and STAT1 phosphorylation without affecting the JNK or p38 MAPK pathway [91]. The evidence in humans remains limited but informative. A randomized prospective study reported short-term improvements in plaque and bleeding indices and a reduced prevalence of selected “red complex” bacteria when an isorhamnetin-containing herbal tincture was used as an adjunct to non-surgical periodontal therapy. In contrast, the intergroup CAL difference, while statistically significant, was clinically minimal over the short follow-up period [92]. Overall, the available data indicate possible benefits of isorhamnetin-containing formulations, although their clinical significance has yet to be clearly established.

Biochanin A. Biochanin A, an isoflavone derived from red clover (*Trifolium pratense*) and peanuts (*Arachis hypogaea*), exhibits antioxidant, anti-inflammatory, and bone-protective effects. It reduces IL-1β and TNF-α levels, suppresses ROS production and osteoclast formation, and enhances osteocalcin expression and bone volume through the activation of Nrf2. In a ligature-induced periodontitis model, Biochanin A treatment markedly alleviated gingival inflammation, oxidative stress, and alveolar bone loss, with increased Nrf2-positive cells and decreased TRAP-positive osteoclasts [93]. Nonetheless, the evidence remains limited to animal models, and dose–response and safety considerations should be interpreted cautiously.

Hesperetin. Hesperetin, a citrus-derived flavanone abundant in citrus fruits (Rutaceae), suppresses osteoclastogenesis and bone resorption by inhibiting NF-κB/MAPK activation, reducing ROS, and downregulating osteoclast markers (TRAP, cathepsin K, NFATc1), while simultaneously activating Nrf2/HO-1 to enhance antioxidant defenses [94]. In vivo, it reduced bone loss and osteoclast numbers and decreased the RANKL/OPG ratio [94]. Taken together, these findings support redox-inflammation coupling in experimental settings, while the clinical evidence specific to periodontitis remains limited.

Epigallocatechin-3-gallate (EGCG). EGCG, the major green tea catechin, exerts antioxidant and anti-inflammatory effects in periodontal disease. In ligature-induced rat periodontitis, systemic EGCG administration reduced alveolar bone loss and inflammatory infiltration, decreased IL-6 and TNF-α expression, and suppressed osteoclast formation [95]. In *Porphyromonas gingivalis*-infected mice, EGCG attenuated alveolar bone resorption and downregulated pro-inflammatory cytokines (IL-1β, IL-6, TNF-α, RANKL, and CCL2), thereby suppressing osteoclastogenesis [96]. Recent data indicate that EGCG upregulates the Nrf2/HO-1 pathway, inhibits NF-κB and NLRP3 activation, reduces IL-1β, IL-18, TNF-α, and MDA levels, and enhances SOD activity in periodontal tissues [97]. Importantly, human clinical evidence suggests modest and context-dependent benefits. In a randomized clinical trial, adjunctive use of EGCG during scaling and root planing showed modest clinical benefits [98]. In contrast, another randomized trial reported improvements in bleeding indices, with limited effects on other periodontal parameters [99]. Overall, the available clinical data suggest that EGCG provides modest adjunctive benefits in periodontitis, although the reported outcomes vary across studies.

Chlorogenic acid (CA). CA, abundant in coffee and several plant foods, exerts antioxidant, anti-inflammatory, and anti-resorptive effects via multiple signaling pathways. It decreases IL-1β, IL-18, and ROS levels, promotes Nrf2 nuclear translocation and HO-1 expression, and suppresses CysLT1R, NLRP3, and NF-κB signaling [100]. In *Porphyromonas gingivalis* LPS-stimulated human gingival fibroblasts, CA markedly attenuated inflammatory responses by suppressing the TLR4/MyD88–NF-κB and PI3K/MAPK signaling pathways, thereby reducing iNOS, COX-2, NO, and PGE_2_ expression [101]. CA inhibits RANKL-induced osteoclast differentiation in bone marrow macrophages by suppressing the NF-κB and MAPK signaling pathways, thereby reducing NFATc1, TRAP, and OSCAR expression and attenuating LPS-induced bone resorption in vivo [102]. In addition, a PLGA/PVP-based nanocarrier enabling sustained local delivery of CA effectively prolonged tissue retention and alleviated inflammation and alveolar bone resorption [103].

Astaxanthin. Astaxanthin, a xanthophyll carotenoid found in microalgae and seafood, attenuates ligature-induced periodontal inflammation and alveolar bone loss by decreasing osteoclast numbers and enhancing osteoblast activity and osteocalcin expression [104]. In ovariectomized osteoporotic mice, oral administration of astaxanthin restored trabecular bone volume and mineral density, inhibited serum TRAP activity, and downregulated RANKL-induced expression of NFATc1, TRAP, DC-STAMP, and cathepsin K in bone marrow macrophages [105]. In parallel, astaxanthin activated Nrf2/HO-1 signaling, restored SOD and CAT activity, and alleviated oxidative stress and pro-inflammatory mediator expression in periodontal tissues and AGE-treated hPDLCs [106].

Sinensetin (Bach1-targeting phytochemical). Sinensetin, a citrus-derived polymethoxylated flavone, represents a mechanistically distinct subclass of dietary phytochemicals. It directly binds to the transcriptional repressor Bach1, promotes its ubiquitination-dependent degradation, and relieves the Bach1-mediated repression of HMOX1, thereby enhancing HO-1 expression and attenuating oxidative stress and inflammation in periodontitis models [53].

In addition, melatonin, an endogenous indoleamine with well-established antioxidant and anti-inflammatory properties, has been reported to modulate Nrf2/HO-1-related signaling in experimental models [107]; however, its periodontal relevance has been explored mainly in the context of engineered delivery systems, such as melatonin-derived carbon dots, as discussed in Section 6.4.

### 6.2. Herbal and Traditional Medicine-Derived Redox Modulators Investigated in Experimental Periodontitis

Table 2 lists herbal and traditional medicine-derived compounds that have been reported to modulate oxidative and inflammatory pathways in experimental models of periodontitis. Natural products such as curcumin, magnolol, paeonol, and berberine have been shown to activate Nrf2-related antioxidant responses. These effects are often accompanied by the suppression of NF-κB and MAPK signaling, leading to reduced inflammation, oxidative stress, and osteoclast differentiation. These agents are largely derived from traditional herbal medicines and link traditional use with modern redox-focused periodontal research, although the strength of the evidence varies among compounds.

Curcumin. Curcumin, a polyphenolic compound derived from *Curcuma longa*, exerts antioxidant, anti-inflammatory, and pro-osteogenic effects in periodontal tissues. It enhances alkaline phosphatase activity, mineralization, and osteogenic gene expression in human periodontal ligament stem cells via activation of the PI3K/Akt–Nrf2 signaling pathway [108]. In *Fusobacterium nucleatum*-challenged oral epithelial cells, curcumin suppressed NF-κB activation while promoting Nrf2/HO-1 expression [109]. In vivo, curcumin attenuated *Porphyromonas gingivalis*- and ligature-induced periodontitis by downregulating IL-1β, TNF-α, and RANKL expression, suppressing osteoclastogenesis, and preventing alveolar bone loss [110]. Furthermore, curcumin mitigates ferroptosis in ligature-induced periodontitis by restoring redox homeostasis, elevating SOD and GSH levels, and reducing MDA accumulation. Mechanistically, it upregulates SLC7A11 and GPX4 while downregulating ACSL4 and TfR1, thereby preventing ferroptotic injury and alveolar bone loss [111]. Notably, a few randomized trials and systematic reviews suggest that curcumin, given locally or systemically as an adjunct to conventional therapy, may improve periodontal inflammatory outcomes; however, the evidence in humans remains limited and heterogeneous [89,112].

Magnolol. Magnolol, a bioactive lignan from *Magnolia officinalis*, attenuates periodontal inflammation and alveolar bone resorption through antioxidant, anti-inflammatory, and antiresorptive effects. It activates the p38 MAPK–ROS–Nrf2/HO-1 signaling cascade, thereby suppressing *Porphyromonas gingivalis* LPS-induced cytokine production, including IL-1β and TNF-α, and inhibiting NF-κB activation [113]. In ligature-induced periodontitis, magnolol attenuated neutrophil infiltration, oxidative stress, and the expression of iNOS, COX-2, and MMP-1/9, while downregulating RANKL expression, reducing osteoclast formation, and ameliorating alveolar bone loss [114]. To date, evidence for magnolol in periodontitis is largely limited to experimental models, and clinical data in humans are not yet available.

Paeonol. Paeonol, a phenolic compound derived from Moutan Cortex, exerts anti-inflammatory, antioxidant, and anti-resorptive effects in periodontal disease. It attenuates alveolar bone resorption, suppresses pro-inflammatory cytokines including IL-1β, IL-6, and TNF-α, and alleviates oxidative stress through activation of the Nrf2/HO-1 pathway and inhibition of NF-κB and NFATc1 signaling [76]. In experimental models, paeonol administration reduced osteoclast formation, inflammatory infiltration, and alveolar bone loss induced by lipopolysaccharide or ligature challenge. Radiographic and histological analyses confirmed the preservation of periodontal bone architecture and downregulation of IL-1β, IL-6, and TNF-α expression in gingival tissues [115]. At present, the evidence for paeonol in periodontitis is primarily derived from animal studies, and clinical data in humans are lacking.

Resveratrol. Resveratrol, a natural polyphenol with potent antioxidant and anti-inflammatory activity, suppresses NF-κB signaling and its downstream targets (COX-2, MMP-2, MMP-9, TLR4), while restoring Nrf2/HO-1 expression and reducing ROS accumulation in human gingival fibroblasts [116]. Resveratrol enhances the osteogenic differentiation of periodontal ligament stem cells through activation of the Nrf2/HO-1 pathway and inhibition of NF-κB signaling [117]. In vivo, oral administration of resveratrol attenuated alveolar bone loss, suppressed osteoclastogenesis, and restored antioxidant defense systems. In ligature-induced rat periodontitis models, resveratrol ameliorated alveolar bone resorption and reduced oxidative and nitrosative stress through activation of the Sirt1/AMPK and Nrf2/antioxidant defense pathways [118]. Similarly, the systemic administration of resveratrol reduced the IL-1β and IFN-γ levels in gingival tissues and significantly preserved the alveolar bone structure in experimental periodontitis [119]. Local delivery of resveratrol further promoted bone regeneration by downregulating TNF-α and IL-6 and enhancing angiogenic (CD31) and osteogenic (OCN, RUNX2) markers [120]. In addition, several clinical studies and systematic reviews have suggested that resveratrol supplementation may improve periodontal inflammatory parameters when used as an adjunct to conventional therapy; however, the available evidence in humans remains limited and heterogeneous in terms of the study design, dosage, and patient populations [121,122].

Schisandrin. Schisandrin, a natural compound derived from the fruit of *Schisandra chinensis*, inhibits NF-κB nuclear translocation and reduces TNF-α, IL-1β, and IL-6 in RAW264.7 macrophages, largely through HO-1 induction. It promotes Nrf2 nuclear translocation and HO-1 Via PI3K/Akt and ERK pathways [123].

Ginsenoside Rg1. Ginsenoside Rg1, the principal bioactive saponin of *Panax ginseng*, exerts antioxidative and anti-inflammatory effects by activating the AMPK/Nrf2/HO-1 signaling axis in periodontal ligament cells [124]. In ligature- and *Porphyromonas gingivalis*-induced periodontitis, Rg1 downregulated Keap1 while upregulating Nrf2, TGF-β1, RUNX2, and osteocalcin, thereby reducing IL-6 expression, suppressing osteoclastogenesis, and improving alveolar bone microarchitecture [59].

Silibinin. Silibinin, a flavonoid derived from *Silybum marianum*, exhibits strong antioxidant, anti-inflammatory, and osteoprotective effects. In a ligature-induced periodontitis model, silibinin significantly reduced alveolar bone loss and gingival inflammation, while suppressing NF-κB and NLRP3 activation and maintaining Nrf2 expression [125]. In vitro, silibinin inhibited RANKL-induced osteoclast differentiation and NFATc1 activation through NF-κB signaling and suppressed inflammatory cytokine production and oxidative stress in LPS-stimulated human gingival fibroblasts (HGFs) [126]. In coculture systems of HGFs and monocytes, silibinin disrupted osteoclastogenesis and attenuated inflammation-driven osteoclast activation, supporting its potential role in modulating HGF-mediated osteoclastogenic responses [126].

Sappanchalcone. Sappanchalcone, a flavonoid isolated from *Caesalpinia sappan*, exerts antioxidant, anti-inflammatory, and osteoprotective effects. Sappanchalcone induces HO-1 expression via JNK-dependent Nrf2 nuclear translocation, protecting dental pulp and periodontal ligament cells (PDLCs) from oxidative and inflammatory injury [127]. It downregulated iNOS, COX-2, NO, and PGE_2_, as well as cytokines including IL-1β, IL-6, and TNF-α, effects that were reversed by HO-1 inhibition [127]. Recent evidence shows that Sappanchalcone enhances PDLC osteogenic differentiation, increasing Runx2, osteopontin, and ALP expression, and promoting mineralized nodule formation [128]. In vivo, Sappanchalcone reduced alveolar bone resorption and improved fiber alignment and junctional epithelium proliferation in rat periodontitis models [128].

### 6.3. Small Molecules, Lipid-Derived Mediators, and Microbial Redox Metabolites Investigated in Experimental Periodontitis

As summarized in Table 3, several novel small molecules, including lipid-derived and microbial metabolites such as itaconate, 4-octyl itaconate, 10-oxo-trans-11-octadecenoic acid (KetoC), and nitro-oleic acid, exert antioxidant and anti-inflammatory effects through the activation of Nrf2 and the inhibition of NF-κB signaling.

Most of the small molecules and redox-active metabolites discussed in this section are currently available as research-grade compounds and have been primarily investigated in vitro or in animal models. Several agents, such as four-octyl itaconate and nitro-oleic acid, represent chemically modified or stabilized derivatives designed to improve the cellular permeability or bioactivity, whereas others, including microbial-derived metabolites, are endogenously generated molecules examined mainly in experimental settings. At present, these compounds are not approved for clinical use in periodontitis, and their biological effects are highly dependent on the formulation, dosing, and delivery strategies.

Four-octyl itaconate (4-OI). 4-OI, a cell-permeable derivative of itaconate, ameliorates ligature-induced periodontal inflammation and alveolar bone loss via activation of Nrf2 signaling [57]. It promotes macrophage polarization from M1 to M2, suppresses IL-6, IL-1β, and iNOS, and scavenges excessive ROS [57]. Mechanistically, 4-OI alkylates KEAP1 cysteine residues and disrupts the Keap1–Nrf2 complex, leading to Nrf2 release and the induction of downstream antioxidant enzymes such as HO-1, NQO1, PRDX1, and GCLM; these effects were abolished in Nrf2-deficient mice [57].

10-oxo-trans-11-octadecenoic acid (KetoC). KetoC, a gut microbiota-derived fatty acid metabolite of linoleic acid, exerts anti-inflammatory and antioxidant effects. In *Porphyromonas gingivalis* LPS-stimulated macrophages, KetoC suppressed TNF-α, IL-6, and IL-1β expression by inhibiting NF-κB p65 nuclear translocation via activation of the G-protein-coupled receptor GPR120 [129]. In gingival epithelial cells, KetoC promoted Nrf2 nuclear translocation and ARE binding, upregulating HO-1 and NQO1 and reducing ROS levels through the GPR120–ERK–Nrf2 signaling axis [130].

Lindenenyl acetate. Lindenenyl acetate, a sesquiterpene from *Lindera strychnifolia*, exhibits potent anti-inflammatory effects in *Porphyromonas gingivalis* LPS-stimulated human periodontal ligament cells. Lindenenyl acetate suppresses iNOS-derived NO, COX-2-mediated PGE_2_, and pro-inflammatory cytokines including IL-1β, TNF-α, IL-6, and IL-12 [131]. It induces Nrf2 nuclear translocation and upregulates HO-1 expression and activity; inhibition of HO-1 by tin protoporphyrin abolished these effects [131]. Lindenenyl acetate also activates JNK and AMPK signaling and enhances the phosphorylation of LKB1 and CaMKII, while AMPK inhibition reverses its anti-inflammatory actions [131].

Nootkatone. Nootkatone, a bioactive sesquiterpenoid found in grapefruit and *Alaska cedar*, attenuates periodontal inflammation and alveolar bone loss through activation of the Nrf2/HO-1 pathway and inhibition of NF-κB signaling [79]. In a ligature-induced periodontitis rat model, oral administration of nootkatone reduced the CEJ–ABC distance and improved the bone mineral density, bone volume, and the BV/TV ratio. Histological analyses revealed decreased osteoclast numbers, restoration of the gingival architecture, and reduced IL-1β, IL-6, and TNF-α expression in gingival tissues [79].

Euphorbia factor L1. Euphorbia factor L1 (EFL1), a lathyrane-type diterpenoid isolated from *Euphorbia lathyris*, modulates osteoclastogenesis and redox signaling through regulation of the NF-κB and Nrf2 pathways [132]. EFL1 inhibits RANKL-induced osteoclast formation by suppressing NF-κB, c-Fos, and NFATc1 signaling while upregulating Nrf2 and HO-1, thereby reducing ROS production. It also suppresses PGC-1β-dependent mitochondrial biogenesis and promotes Fas/FasL-mediated apoptosis in mature osteoclasts [132]. To date, the evidence for EFL1 in periodontitis is limited, with the reported effects primarily derived from experimental models of inflammatory bone loss.

Dehydrocostus lactone. Dehydrocostus lactone (DL), a natural sesquiterpene lactone, suppresses RANKL-induced osteoclastogenesis by inhibiting NF-κB, AP-1, and NFATc1 signaling via regulation of IKK and JNK, while activating Nrf2 [133]. DL reduces ROS through both direct scavenging and Nrf2-dependent antioxidant responses. It decreases the phosphorylation of IκBα and p65, suppresses c-Fos and JNK activation, and upregulates antioxidant enzymes such as Srx and Prx1. The inhibitory effect on NFATc1 and osteoclast formation was markedly reduced in Nrf2-deficient cells [133].

### 6.4. Nanomaterials and Redox-Responsive Delivery Platforms Investigated in Experimental Periodontitis

Table 4 summarizes nanomaterials and redox-responsive delivery systems developed to achieve sustained Nrf2 activation and ROS scavenging within periodontal tissues. Platforms such as N-acetyl-L-cysteine-derived carbon dots, melatonin-based nanomaterials, and redox-responsive hydrogels enable localized antioxidant delivery and support tissue repair in experimental periodontitis models. These nanosystems bridge materials science and molecular redox biology, providing a foundation for controlled antioxidant and regenerative delivery in periodontitis.

NAC-derived carbonized polymer dots (NAC-CPDs). NAC-CPDs, synthesized from N-acetyl-L-cysteine via a hydrothermal process, show good biocompatibility and ROS-scavenging capacity. They restore the redox balance, promote osteogenic differentiation of human periodontal ligament cells under oxidative stress, and selectively accumulate in alveolar bone in vivo. In a mouse periodontitis model, NAC-CPDs reduced bone resorption and inflammation through Keap1/Nrf2-dependent antioxidant signaling [134].

Melatonin-derived carbon dots (MT-CDs). MT-CDs exhibit good water solubility and biocompatibility and demonstrate enhanced ROS-scavenging activity compared with native melatonin. They reduce intracellular ROS, preserve mitochondrial homeostasis, and suppress pro-inflammatory mediators in vitro. In a mouse periodontitis model, MT-CDs inhibited alveolar bone loss, osteoclast activation, and inflammatory infiltration while promoting tissue repair through activation of the Nrf2/HO-1 pathway [107].

Cordycepin-loaded microspheres (MMS-CY). MMS-CY, adhesive and mineralized hydrogel microspheres delivering cordycepin, enhance periodontal ligament stem cell (PDLSC) migration, proliferation, and osteogenic differentiation under inflammatory conditions. They activate Nrf2 signaling, upregulate NQO1, and suppress senescence-associated genes (IL-6, IL-8) while reducing DNA damage in PDLSCs [78]. In a ligature-induced rat periodontitis model, MMS-CY improved collagen fiber organization, decreased osteoclast activity, and promoted alveolar bone regeneration [78].

## 7. Diabetes-Associated Periodontitis and Nrf2 Dysfunction

Diabetes mellitus (DM) is a well-established systemic risk factor that accelerates the onset and severity of periodontitis. Hyperglycemia impairs innate immune responses, induces microvascular dysfunction, delays wound healing, and promotes the accumulation of advanced glycation end-products (AGEs). Engagement of AGEs with their receptor (RAGE) amplifies oxidative and inflammatory signaling cascades [135]. Conversely, chronic periodontal inflammation elevates circulating cytokines such as TNF-α and IL-6, contributing to insulin resistance and impaired glycemic control [136,137]. While this bidirectional relationship underscores the interplay between metabolic dysregulation and periodontal inflammation, the contribution of oxidative stress-responsive pathways, including Nrf2 signaling, likely differs according to the disease stage and metabolic condition.

Large cohort studies, including the Health Professionals Follow-Up Study, demonstrate that type 2 diabetes mellitus (T2DM) significantly increases the susceptibility to periodontitis and tooth loss, particularly in individuals with low dietary antioxidant intake [138]. Clinical biomarker studies further reveal a reduced total antioxidant capacity (TAOC) and elevated oxidative stress index (OSI) in gingival crevicular fluid (GCF), correlating with periodontal pocket depth and attachment loss [139]. Patients with T2DM and periodontitis present higher GCF AGE levels than non-diabetic individuals, with positive associations with HbA1c levels and marginal bone loss [140]. Increased systemic and local oxidative stress—reflected by elevated reactive oxygen metabolites and decreased SOD and GPx activity—has been associated with impaired Nrf2/HO-1 signaling in diabetes-associated periodontitis [141,142].

Mechanistic studies identify oxidative stress and mitochondrial dysfunction as shared pathogenic drivers of diabetes and periodontitis [143]. Hyperglycemia induces excessive mitochondrial ROS generation, impairing insulin signaling, promoting NF-κB-dependent inflammation, and triggering apoptosis in periodontal vascular and stromal cells [144,145,146,147]. Mitochondrial DNA damage, reduced mitochondrial mass, and impaired oxidative phosphorylation have been documented in diabetic patients with periodontitis, reinforcing the convergence of metabolic and inflammatory stress [148]. In streptozotocin-induced diabetic rats with ligature-induced periodontitis, alveolar bone loss, oxidative injury, and periodontal apoptosis are markedly exacerbated, accompanied by reduced Nrf2 expression and antioxidant enzyme activity [15]. However, most of these findings are derived from experimental or cross-sectional studies; therefore, they could not determine whether impaired Nrf2 signaling acts as a primary driver or arises secondary to chronic metabolic and inflammatory stress in diabetes-associated periodontitis.

The sirtuin family—particularly SIRT1, SIRT3, and SIRT6—interacts with Nrf2 signaling to regulate mitochondrial integrity and glucose metabolism [149]. SIRT1 enhances insulin sensitivity and β-cell survival through the deacetylation of Nrf2 and FOXO1 [150,151]. SIRT3 maintains the mitochondrial redox balance via activation of SOD2 and GPx1 [152,153], while SIRT6 supports DNA repair and suppresses NF-κB-driven inflammation [154,155]. Although these interactions point to a coordinated antioxidant regulatory axis, the available evidence is largely derived from experimental models, and their relevance to human diabetic periodontitis has not been fully established.

Recent research highlights the therapeutic potential of targeting Nrf2 to counteract hyperglycemia-induced oxidative stress. Natural compounds such as albiflorin enhance cell viability and reduce ROS in AGE-stimulated gingival fibroblasts via Nrf2 activation and NF-κB inhibition [156]. Baicalein restores mitochondrial homeostasis, activates Nrf2, and reduces alveolar bone loss in diabetic periodontitis [157]. Magnolol attenuates oxidative stress and cytokine production in AGE-stimulated fibroblasts through Nrf2/HO-1 activation [158]. Beyond phytochemicals, the Ganoderma immunomodulatory protein (GMI) activates Nrf2 and suppresses NF-κB in AGE/LPS-challenged fibroblasts [159]. Additionally, exosomal microRNA-141-3p from periodontal ligament stem cells suppresses KEAP1 and enhances Nrf2 activity, alleviating high-glucose-induced senescence [160]. The probiotic *Clostridium butyricum* MIYAIRI 588 reduces alveolar bone loss in diabetic mice by restoring the gut microbial balance and elevating 4-hydroxybenzenemethanol, a microbiota-derived Nrf2 activator [161]. Notably, most findings are derived from in vitro or animal models, and issues related to bioavailability, dosing, and long-term safety remain unresolved in diabetes-associated periodontitis. Representative therapeutic agents targeting Nrf2 in diabetic periodontitis are summarized in Table 5.

Overall, the available evidence indicates that diabetes is associated with exacerbated redox imbalance and inflammatory injury in periodontal tissues, accompanied by alterations in Nrf2 signaling, mitochondrial homeostasis, and sirtuin activity. Strategies aimed at restoring Nrf2 activation, improving mitochondrial function, and modulating gut-derived redox metabolites may represent a potential host-modulation approach for diabetes-associated periodontitis. Further translational studies and well-designed clinical trials are required to clarify the clinical relevance of Nrf2-based therapeutic strategies in this patient population.

## 8. Conclusions and Future Perspectives

Periodontitis is driven by persistent oxidative stress and dysregulated inflammatory signaling, in which impaired Nrf2 activity contributes to tissue destruction and alveolar bone loss. Activation of the Nrf2 pathway restores the antioxidant capacity, suppresses NF-κB- and NLRP3-mediated inflammation, and promotes osteogenic repair, positioning Nrf2 as an important regulatory node linking redox imbalance and bone remodeling. Nevertheless, whether Nrf2 dysfunction represents a primary driver of disease progression or a secondary consequence of chronic inflammation and microbial dysbiosis remains incompletely understood. Further, the translation of Nrf2-targeted strategies into clinical care remains limited. Future work should prioritize standardized dosing and optimized delivery systems. In particular, nanoparticle- or hydrogel-based sustained-release formulations should be evaluated in preclinical and clinical models. Integrating Nrf2 modulation with metabolic control, microbiome regulation, and personalized therapeutic approaches may enable precision host-modulatory therapy. Although antioxidant toothpastes containing polyphenolic or herbal compounds have demonstrated anti-inflammatory effects in experimental periodontal models, direct evidence linking these formulations to Nrf2 pathway activation in periodontal tissues is currently lacking. Addressing the variability across experimental models and the paucity of human interventional data will be critical for clarifying the therapeutic scope of Nrf2-centered strategies. Advancing these strategies through rigorously designed preclinical and clinical studies will be essential to fully realize the therapeutic potential of Nrf2-centered interventions in periodontal and systemic health.

## Figures and Tables

**Table 1:** Representative Redox-Active Dietary Phytochemicals Investigated in Experimental Models of Periodontitis.

Modulator	Source	Experimental Model	Mechanism/Pathway	Key Effects	Evidence Level
Sulforaphane	Isothiocyanate (Cruciferous vegetables)	In vitro (glutathione synthesis inhibitor-treated human neutrophils, LPS-stimulated human gingival epithelial cells (HGFs))In vivo (ligature-induced periodontitis rat model)	Activates ERK–Nrf2–ARE	↓ROS ↓NADPH oxidase ↓IL-6, ↓TNF-α, ↓COX-2 ↓Osteoclast ↓Alveolar bone loss	Animal Cell
Quercetin	Flavonoid (fruits, vegetables)	In vitro (H_2_O_2_-stimulated human periodontal ligament cells (hPDLCs)) In vivo (ligature-induced periodontitis mouse and rat models, *A. actinomycetemcomitans*-induced periodontitis mouse model)	Activates Nrf2/HO-1 Modulates p53/p21	↓IL-1β, ↓TNF-α ↓iNOS, ↓MMP-8, ↓Apoptosis ↓Senescence ↓Alveolar bone loss	Human (limited) Animal Cell
Isorhamnetin	Flavonoid (*Hippophae rhamnoides*)	In vitro (LPS-stimulated HGFs, LPS-stimulated RAW264.7)	Activates Nrf2/HO-1 Inhibits NF-κB	↓IL-6, ↓IL-8 ↓PGE_2_, ↓NO	Human (limited) Cell
Biochanin A	Isoflavone (red clover, peanuts)	In vitro (Biochanin A-treated rat gingival fibroblasts)In vivo (ligature-induced periodontitis rat model)	Activates Nrf2	↓IL-1β, ↓TNF-α, ↓ROS ↑Osteocalcin ↓Alveolar bone volume	Animal Cell
Hesperetin	Flavanone (citrus fruits)	In vitro (LPS-stimulated RAW264.7 cells) In vivo (LPS-induced periodontitis rat model)	Activates Nrf2/HO-1 Inhibits NF-κB/MAPK	↓ROS ↓Osteoclastogenesis ↓RANKL/OPG ratio ↓Alveolar bone resorption	Animal Cell
EGCG	Catechin (green tea)	In vivo (ligature-induced periodontitis rat model, *Porphyromonas gingivalis*-induced periodontitis mouse model)	Activates Nrf2/HO-1 Inhibits NF-κB and NLRP3 inflammasome	↓IL-1β, ↓IL-6, ↓TNF-α ↑SOD, ↓MDA ↓Alveolar bone loss	Human Animal Cell
Chlorogenic acid	Polyphenol (coffee, fruits)	In vitro (LPS-stimulated HGFs, RANKL-stimulated mouse bone marrow macrophages, LPS-stimulated RAW264.7 cells) In vivo (LPS-induced periodontitis mouse and rat models)	Activates Nrf2/HO-1 Suppresses TLR4/MyD88-NF-κB, NLRP3	↓ROS ↓IL-1β, ↓IL-18 ↓iNOS, ↓NO, ↓COX-2 ↓Osteoclastogenesis ↓Alveolar bone loss	Animal Cell
Astaxanthin	Carotenoid (marine algae)	In vitro (RANKL-stimulated mouse bone marrow cells, AGE-stimulated hPDLCs) In vivo (ligature-induced periodontitis rat model, ovariectomized mice, STZ-induced diabetic periodontitis mouse model)	Activates Nrf2/HO-1	↑SOD, ↑CAT ↑Osteoblast, ↓Osteoclast ↓Alveolar bone loss	Animal Cell
**Sinensetin**	Polymethoxylated flavone (citrus-derived)	In vitro (TNF-α and IL-1β-stimulated hPDLCs) In vivo (ligature-induced periodontitis rat model)	Degrades Bach1 Induces HO-1	↓ROS, ↓MDA ↓IL-6, ↑IL-10 ↓Alveolar bone loss	Animal Cell

The evidence level indicates the level of experimental support reported for each compound in periodontitis-related studies (Human, Animal, Cell).

**Table 2:** Herbal and Traditional Medicine-Derived Redox Modulators Investigated in Experimental Models of Periodontitis.

Modulator	Source	Experimental Model	Mechanism/Pathway	Key Effects	Evidence Level
Curcumin	Polyphenol (*Curcuma longa*)	In vitro (human periodontal ligament stem cells (hPDLSCs) under osteogenic induction, *Fusobacterium nucleatum*-stimulated human oral epithelial cells) In vivo (*Porphyromonas gingivalis*- and ligature-induced periodontitis rat model, ligature-induced periodontitis mouse model)	Activates Nrf2/HO-1 and PI3K/Akt Suppresses NF-κB	↑ALP, ↑Mineralization ↓IL-1β, ↓TNF-α, ↓RANKL ↓Osteoclastogenesis ↓Alveolar bone loss ↓Ferroptosis	Human (limited) Animal Cell
Magnolol	Neolignan (*Magnolia officinalis*)	In vitro (LPS-stimulated RAW264.7 cells, RANKL-stimulated RAW264.7 cells) In vivo (Ligature-induced periodontitis rat model)	Activates p38 MAPK–ROS–Nrf2/HO-1 axis Inhibits NF-κB	↓IL-1β, ↓TNF-α ↓iNOS, ↓COX-2 ↓Osteoclastogenesis ↓Alveolar bone loss	Animal Cell
Paeonol	Phenolic compound (Moutan Cortex)	In vitro (RANKL-stimulated RAW264.7 cells) In vivo (LPS- and ligature-induced periodontitis rat model)	Activates Nrf2/HO-1 Inhibits NF-κB and NFATc1	↓IL-1β, ↓IL-6, ↓TNF-α ↓Osteoclast formation ↓Alveolar bone loss	Animal Cell
Resveratrol	Polyphenol (grapes, berries)	In vitro (LPS-stimulated HGFs, LPS-stimulated hPDLSCs) In vivo (LPS- and ligature-induced periodontitis rat model, ligature-induced periodontitis mouse and rat models)	Activates Nrf2/HO-1 and Sirt1/AMPK Inhibits NF-κB	↓ROS ↓IL-1β ↓TNF-α ↓Osteoclastogenesis ↑Osteogenesis ↓Alveolar bone loss	Human (limited) Animal Cell
Schisandrin	Lignan (*Schisandra chinensis*)	In vitro (LPS-stimulated RAW264.7 cells)	Activates Nrf2/HO-1 via PI3K/Akt and ERK Inhibits NF-κB	↓IL-1β, ↓IL-6 ↓TNF-α	Cell
Ginsenoside Rg1	Triterpenoid saponin (*Panax ginseng*)	In vitro (LPS-stimulated hPDLCs) In vivo (*Porphyromonas gingivalis*- and ligature-induced periodontitis rat model)	Activates Nrf2/HO-1 Downregulates Keap1	↓IL-6 ↑TGF-β1, ↑RUNX2 ↑Osteocalcin ↓Osteoclast ↓pyroptosis ↓Alveolar bone loss	Animal Cell
Silibinin	Flavonoid (*Silybum marianum*)	In vitro (LPS-stimulated hPDLCs, LPS-stimulated HGFs, RANKL-stimulated RAW264.7 cells) In vivo (ligature-induced periodontitis rat model)	Upregulates Nrf2 Inhibits NF-κB and NLRP3	↓PDLCs apoptosis ↓Osteoclastogenesis ↓Alveolar bone loss	Animal Cell
Sappanchalcone	Flavonoid (*Caesalpinia sappan*)	In vitro (H_2_O_2_-stimulated human dental pulp cells, LPS-stimulated hPDLCs, hPDLCs under osteogenic induction) In vivo (ligature-induced periodontitis rat model)	Induces HO-1 via JNK-dependent Nrf2 nuclear translocation	↓IL-1β, ↓IL-6, ↓TNF-α ↓iNOS, ↓NO ↓PGE_2_, ↓COX-2 ↑RUNX2, ↑ALP ↓Alveolar bone loss	Animal Cell

The evidence level indicates the level of experimental support reported for each compound in periodontitis-related experimental studies (Human, Animal, or Cell).

**Table 3:** Small Molecules, Lipid-Derived Mediators, and Microbial Redox Metabolites Investigated in Experimental Models of Periodontitis.

Modulator	Source	Experimental Model	Mechanism/Pathway	Key Effects	Evidence Level
Four-octyl itaconate (4-OI)	Itaconate derivative	In vitro (LPS-stimulated RAW264.7 cells) In vivo (ligature-induced periodontitis mouse model)	Alkylates KEAP1; disassociates Keap1-Nrf2 complex	↓IL-1β, ↓IL-6, ↓iNOS ↑HO-1, ↑NQO1, ↓ROS ↑M2 polarization ↓Alveolar bone loss	Animal Cell
10-oxo-trans-11-octadecenoic acid (KetoC)	Microbial metabolite	In vitro (LPS-stimulated RAW264.7 cells, Tert-butyl hydroperoxide-stimulated human gingival epithelial cells)	Activates GPR120–ERK–Nrf2 axis Inhibits NF-κB	↓IL-1β, ↓IL-6, ↓TNF-α ↑HO-1, ↑NQO1, ↓ROS	Cell
Lindenenyl acetate	Sesquiterpene (*Lindera strychnifolia*)	In vitro (LPS-stimulated hPDLCs)	Activates Nrf2/HO-1 Activates JNK/AMPK	↓IL-1β, ↓IL-6, ↓TNF-α ↓iNOS/NO, ↑HO-1 ↓COX-2/PGE_2_	Cell
Nootkatone	Sesquiterpenoid (grapefruit, *Alpinia* spp.)	In vivo (ligature-induced periodontitis rat model)	Activates Nrf2/HO-1 Inhibits NF-κB	↓IL-1β, ↓IL-6, ↓TNF-α ↓MDA, ↑SOD, ↓Osteoclast number ↓Alveolar bone loss	Animal
Euphorbia factor L1	Diterpenoid (*Euphorbia lathyris*)	In vitro (RANKL-stimulated mouse bone marrow-derived macrophages) In vivo (ovariectomy-induced bone loss mouse model)	Activates Nrf2 Inhibits NF-κB/c-Fos/NFATc1 and PGC-1β	↓ROS, ↑NQO1 ↑Osteoclast apoptosis ↓Osteoclastogenesis	Animal Cell
Dehydrocostus lactone	Sesquiterpene lactone (*Saussurea* spp.)	In vitro (RANKL-stimulated RAW264.7 cells)	Inhibits NF-κB/AP-1/NFATc1 Activates Nrf2	↓IL-1β ↓ROS, ↑NQO1 ↓Osteoclastogenesis	Cell

The evidence level indicates the level of experimental support reported for each compound in periodontitis-related experimental studies (Human, Animal, or Cell).

**Table 4:** Nanomaterials and Redox-Responsive Delivery Platforms Investigated in Experimental Models of Periodontitis.

Modulator	Source	Experimental Model	Mechanism/Pathway	Key Effects	Evidence Level
NAC-derived carbonized polymer dots (NAC-CPDs)	Polymer nanodots	In vitro (H_2_O_2_-stimulated hPDLCs) In vivo (periodontitis mouse model)	Modulates Keap1/Nrf2	↓ROS ↑Osteogenesis ↓Alveolar bone loss	Animal Cell
Melatonin-derived carbon dots (MT-CDs)	Carbon nanodots	In vivo (periodontitis mouse model)	Modulates Nrf2/HO-1	↓ROS ↓Inflammation ↓Osteoclasts ↓Alveolar bone loss	Animal
Cordycepin-loaded microspheres (MMS-CY)	Microsphere formulation	In vitro (LPS-stimulated hPDLSCs, MMS-CY-treated RAW264.7 cells) In vivo (ligature-induced periodontitis rat model)	Activates Nrf2 pathway	↑NQO1, ↑GCLM ↓Senescence ↑Migration ↑Ligament-forming ↑Osteogenesis ↓Osteoclastogenesis ↓Alveolar bone loss	Animal Cell

The evidence level indicates the level of experimental support reported for each platform in periodontitis-related studies, primarily based on animal or cell models.

**Table 5:** Nrf2-Related Modulators Investigated in Experimental Models of Diabetes-Associated Periodontitis.

Modulator	Source	Experimental Model	Mechanism/Pathway	Key Effects	Evidence Level
Albiflorin	Monoterpene glycoside (*Paeonia lactiflora*)	In vitro (AGE-stimulated HGFs)	Modulates Nrf2/NF-κB pathways	↑Cell viability ↓ROS ↓IL-6, ↓IL-8, ↓RAGE	Cell
Baicalein	Flavonoid (*Scutellaria baicalensis*)	In vitro (LPS-stimulated HGFs) In vivo (STZ-induced diabetic periodontitis rat model)	Activates Nrf2	↑CAT, ↑SOD1, ↑SOD2 Restores mitochondrial function ↓Alveolar bone loss	Animal Cell
Magnolol	Neolignan (*Magnolia officinalis*)	In vitro (AGE-stimulated HGFs)	Activates Nrf2/HO-1	↓ROS accumulation ↓IL-6, ↓IL-8 ↑Cell migration	Cell
Ganoderma immunomodulatory protein (GMI)	Protein (*Ganoderma microsporum*)	In vitro (AGE- and LPS-stimulated HGFs)	Activates Nrf2/HO-1 Inhibits NF-κB	↓ROS production ↓IL-6, ↓IL-8 ↓Cellular Senescence	Cell
PDLSC-derived exosomes (miR-141-3p)	Stem cell–derived extracellular vesicles	In vitro (high-glucose-treated hPDLSCs)	Downregulates KEAP1 Activates Nrf2/HO-1	↓IL-6, ↓IL-8 ↓Cellular Senescence ↓MDA, ↑SOD	Cell
*Clostridium butyricum* MIYAIRI 588 (CBM588)	Probiotic (gut-derived)	In vivo (STZ-induced diabetic periodontitis rat model)	Activates Nrf2	↑Serum 4-HBA ↓Alveolar bone loss Modulates macrophage polarization	Animal

The evidence level indicates the level of experimental support reported for each modulator in diabetes-associated periodontitis-related studies (Human, Animal, or Cell).

## Data Availability

No new data were created or analyzed in this study. Data sharing is not applicable to this article.

## Abbreviations

The following abbreviations are used in this manuscript:

IL-1β	interleukin-1β
TNF-α	tumor necrosis factor-α
MMPs	matrix metalloproteinases
ROS	reactive oxygen species
Nrf2	nuclear factor erythroid 2–related factor 2
Keap1	Kelch-like ECH-associated protein 1
AREs	antioxidant response elements
HO-1	heme oxygenase-1
NQO1	NAD(P)H: quinone oxidoreductase-1
SOD	superoxide dismutase
CAT	catalase
NF-κB	nuclear factor kappa-light-chain-enhancer of activated B cells
NLRP3	NOD-like receptor family pyrin domain-containing 3
TAOC	total antioxidant capacity
GPx	glutathione peroxidase
MDA	malondialdehyde
GSDMD	gasdermin D
RANKL	receptor activator of nuclear factor-κB ligand
HGFs	human gingival fibroblasts
PDLCs	periodontal ligament cells
DM	diabetes mellitus
AGEs	advanced glycation end-products
RAGE	receptor for advanced glycation end-products
T2DM	type 2 diabetes mellitus
OSI	oxidative stress index
GCF	gingival crevicular fluid
hPDLSCs	human periodontal ligament stem cells
